# Identification and development of implementation strategies: the important role of codesign

**DOI:** 10.3389/frhs.2024.1305955

**Published:** 2024-02-07

**Authors:** Patricia J. van der Laag, Berber G. Dorhout, Aaron A. Heeren, Cindy Veenhof, Di-Janne J. A. Barten, Lisette Schoonhoven

**Affiliations:** ^1^ Julius Center for Health Sciences and Primary Care, Nursing Science, University Medical Center Utrecht, University Utrecht, Utrecht, Netherlands; ^2^ Research Group Innovation of Human Movement Care, Research Centre for Healthy and Sustainable Living, Utrecht University of Applied Sciences, Utrecht, Netherlands; ^3^ Division of Human Nutrition and Health, Wageningen University and Research, Wageningen, Netherlands; ^4^ Department of Rehabilitation, Physical Therapy Science & Sports, University Medical Center Utrecht, Utrecht University, Utrecht, Netherlands; ^5^ Center for Physical Therapy Research and Innovation in Primary Care, Julius Health Care Centers, Utrecht, Netherlands; ^6^ Faculty of Health Sciences, University of Southampton, Southampton, United Kingdom

**Keywords:** implementation, strategies, methodology, lifestyle intervention, older adults, codesign, Implementation Strategy Mapping Method, primary care

## Abstract

**Background:**

To date, implementation strategies reported in the literature are commonly poorly described and take the implementation context insufficiently into account. To unravel the black box of implementation strategy development, insight is needed into effective theory-based and practical-informed strategies. The current study aims to describe the stepwise development of a practical-informed and theory-based implementation strategy bundle to implement ProMuscle, a nutrition and exercise intervention for community-dwelling older adults, in multiple settings in primary care.

**Methods:**

The first four steps of Implementation Mapping were adopted to develop appropriate implementation strategies. First, previously identified barriers to implementation were categorized into the constructs of the Consolidated Framework for Implementation Research (CFIR). Second, the CFIR-ERIC matching tool linked barriers to existing implementation strategies. Behavioral change strategies were added from the literature where necessary. Third, evidence for implementation strategies was sought. Fourth, in codesign with involved healthcare professionals and implementation experts, implementation strategies were operationalized to practical implementation activities following the guidance provided by Proctor et al. These practical implementation activities were processed into an implementation toolbox, which can be tailored to a specific context and presents prioritized implementation activities in a chronological order.

**Results:**

A previous study identified and categorized a total of 654 barriers for the implementation of a combined lifestyle intervention within the CFIR framework. Subsequently, the barriers were linked to 40 strategies. Due to the fact that many strategies impacted multiple barriers, seven overarching themes emerged based on the strategies: assessing the context, network internally, network externally, costs, knowledge, champions, and patient needs and resources. Codesign sessions with professionals and implementation experts resulted in the development of supported and tangible implementation activities for the final 20 strategies. The implementation activities were processed into a web-based implementation toolbox, which allows healthcare professionals to tailor the implementation activities to their specific context and guides healthcare professionals to prioritize implementation activities chronologically during their implementation.

**Conclusion:**

A theory-based approach in combination with codesign sessions with stakeholders is a usable Implementation Strategy Mapping Method for developing a practical implementation strategy bundle to implement ProMuscle across multiple settings in primary care. The next step involves evaluating the developed implementation strategies, including the implementation toolbox, to assess their impact on the implementation and adoption of ProMuscle.

## 1 Background

Implementation science focuses on translating evidence-based programs (EBPs) into practice (1). Methods or techniques that are employed to overcome barriers and enhance the adoption, implementation, sustainment, and scale-up of such EBPs are called implementation strategies (2). Implementation strategies are designed to target barriers at different levels, such as the intervention, recipient, organizational, policy, and professional levels (3). Numerous studies describe theories and taxonomies and present implementation strategies tailored to specific levels (3). Evidence-based, detailed implementation strategies are crucial for the successful implementation of EBPs in daily practice (4). However, most studies lack an adequate description of the strategies and how to match them to barriers, which makes it difficult to select optimal strategies and to understand whether and how strategies could be effective for overcoming barriers and supporting the implementation of EBPs (1, 5).

Notably, it is not expected that every setting has similar barriers for implementation; instead, various combinations of barriers are likely to emerge, which may change over time (6, 7). The lack of guidance makes it challenging to translate the strategies to specific contexts for different EBPs (5, 8). Selecting appropriate implementation strategies and mapping and tailoring them to address the barriers in the specific context require a systematic approach. Using an Implementation Strategy Mapping Method encompasses the implementation practice and results in transparent strategies; this enables researchers or implementers to assess whether the developed strategies align with their specific context.

Today, several Implementation Strategy Mapping Methods guide the process of selecting and developing implementation strategies (8), each containing three general steps. First, determinants that could facilitate or hamper the implementation of an EBP within the local context should be assessed. Second, change methods (e.g., behavioral, organizational, or system change) to address these determinants must be identified. At last, implementation strategies need to be developed or selected that incorporate these change methods (5).

One of the most frequently used Implementation Strategy Mapping Methods is “Implementation Mapping” (9). Implementation Mapping, described by Fernandez et al. (9), addresses the need for a theory-based method to influence determinants for implementation. Nowadays, Implementation Mapping is widely used in implementation science for selecting and developing implementation strategies. Implementation Mapping describes five tasks to select, develop, execute, and evaluate strategies based on existing theory to enhance the alignment between context and implementation (9). The tasks are iterative, involving continual revisiting of previous steps throughout the process to ensure all adopters and implementers, outcomes, determinants, and objectives are addressed.

To enhance the alignment of implementation strategies with the context of EBP implementation, Fernandez (9) emphasized the need to engage stakeholders in a collaborative process at each step of Implementation Mapping (9). The context in which an intervention is implemented plays a significant role in deciding whether a strategy will be effective. Moreover, strategies that align with the context will contribute to improved implementation and adoption of an EBP (1, 10), achieving more contextually adapted strategies. The experiences of stakeholders can complement implementation science expertise and provide valuable information for identifying implementation challenges and developing possible ways to target these challenges. There are different ways to engage stakeholders in the development of implementation strategies. Codesign is a method that seeks to optimize the alignment of implementation strategies with the context. Codesign involves the collaboration of both trained and untrained individuals in the creative design and development process (1).

In the literature, there are hardly any studies that fully and systematically describe the selection and development of implementation strategies following the crucial steps of an Implementation Strategy Mapping Method, including attention to stakeholder engagement in the identification of barriers and in the selection and development of implementation strategies (11). With this study, we aimed to provide a transparent description of the strategy development process for implementing a combined lifestyle intervention across multiple settings in primary care following Implementation Mapping as an Implementation Strategy Mapping Method, ensuring attention to specific contexts by engaging relevant stakeholders throughout the process.

The combined lifestyle intervention is called ProMuscle, which aims to maintain the independence of older adults. ProMuscle is a 12-week program that combines resistance exercise training with dietary consultations to increase the daily protein intake. Over the years, ProMuscle has undergone further development and has shown promising effects on physical functioning, strength, and muscle mass among community-dwelling older adults (12). Given the rapid aging of the population, the implementation of combined lifestyle interventions like ProMuscle holds significant potential in contributing to the maintenance of physical independence among older individuals. Ultimately, this could have a positive effect on the prevalence of chronic diseases and reduce healthcare costs.

Therefore, the current study aims to develop implementation strategies using codesign sessions with relevant stakeholders to facilitate the implementation and adoption of ProMuscle across multiple settings in primary care.

## 2 Methods

### 2.1 Study design

A qualitative inductive, codesign approach was used to develop theory-based and practical-informed strategies that could align with different contexts. The reporting of this study adheres to the Standard for Reporting Implementation Studies (StaRI) checklist (13).

### 2.2 Setting

This study is part of the PUMP-fit study, which is centered on implementing ProMuscle in the Netherlands. The primary objective of the PUMP-fit study is to increase the adoption of ProMuscle by selecting and evaluating theory-based, context-tailored implementation strategies. This study was conducted in the Region Foodvalley in the Netherlands. The Region Foodvalley is a collaboration between eight municipalities, local healthcare organizations, universities, and research institutes. Its target is to provide a better nutritional environment for the residents of the region. Within the Region Foodvalley, more than 200 healthcare professionals (HCPs; including physiotherapists and dieticians) work within primary care settings across eight municipalities.

The implementation strategies were developed for, and in codesign with, these professionals because they are the target population for implementing ProMuscle in primary care.

### 2.3 Participants

Physiotherapists and dieticians working in the Region Foodvalley were recruited through various channels, including the interest list of the PUMP-fit study, social media announcements, calls for participation in newsletters of professionals’ associations, and local initiatives. Healthcare professionals were included if they were physiotherapists or dieticians involved in treating older adults within primary care.

Moreover, implementation experts from the Netherlands were personally invited to participate in this study. Specifically, their involvement aimed to provide input on the conceptualization of implementation strategies.

#### 2.3.1 Sample size

Codesign studies share similarities with focus group studies in qualitative research, as high-quality interactive discussions among the cocreators are pivotal for a successful process. Although qualitative research lacks existing rules regarding recommended sample sizes, recommendations have been made to recruit cohorts of 6–12 participants for focus group studies (14). Considering these factors, a recommendation of 10–12 participants for the codesign process is advised, which may also account for dropouts due to the process being conducted over multiple sessions.

### 2.4 Procedure

In this study, the Implementation Strategy Mapping Method, “Implementation Mapping”, was adopted (9). As this study aims to describe the development of implementation strategies, the first four of the five steps of Implementation Mapping were followed. Due to the variations in primary care settings across the Netherlands, it is expected that the context in which ProMuscle is implemented will present diverse contextual determinants; hence, it is anticipated that the implementation strategies will vary for each setting. Therefore, the involvement of various stakeholders during the whole process was perceived as an essential step to align the strategies with the context. Stakeholder involvement was incorporated in various ways into these steps. The procedures for each step are described below.

#### 2.4.1 Step 1. Identifying barriers and theoretical constructs

A preliminary aspect of the PUMP-fit study was the identification of barriers and facilitators of the implementation of a combined lifestyle intervention. Determinants influencing the implementation of ProMuscle in community care were identified by a recently performed scoping review; detailed descriptions of these determinants can be found elsewhere (15)*.* In short, a literature review, including stakeholder consultation, was conducted to identify determinants influencing the implementation of combined lifestyle interventions for community-dwelling older adults. The identified barriers were categorized into the constructs of the Consolidated Framework for Implementation Research (CFIR) (16). The CFIR consolidates implementation determinants from various implementation theories and comprises five major domains (namely, intervention characteristics, outer setting, inner setting, characteristics of individuals, and process) made up of 39 constructs that influence the implementation of innovations into practice. Eventually, to validate the identified barriers and facilitators in the literature, 19 relevant stakeholders were consulted. During (group) interviews, 13 physiotherapists, 3 dieticians, and 3 community-dwelling older adults were asked about determinants for implementation, eventually prioritizing the identified barriers.

In addition to mapping and prioritizing the determinants described by Implementation Mapping, relevant implementation models addressing behavioral change (17, 18), organizational change (19), and implementation effectiveness (20) were consulted to establish links between the emerged CFIR constructs and the underlying theoretical constructs. By linking determinants to theoretical constructs, relevant theories were identified, allowing for the adoption of uniform definitions. Eventually, this linking of determinants to underlying constructs provides further direction for justifying possible strategies, which is part of the next steps (21).

#### 2.4.2 Step 2. Linking barriers to strategies

Two methods were used to link the identified barriers to implementation strategies. First, existing taxonomies, models, and theories described in the literature were studied to select implementation strategies. After that, stakeholders were consulted to contribute to the development of additional strategies.

##### 2.4.2.1 Linking to existing taxonomies described in the literature

The first taxonomy used to select strategies was the Expert Recommendations for Implementing Change (ERIC) taxonomy (22). The ERIC taxonomy is a widely used compilation of 73 implementation strategies consisting of definitions sourced from a wide range of implementation experts. To link the identified barriers to possible implementation strategies, the CFIR-ERIC Matching Tool was used (5). The CFIR-ERIC Matching Tool was developed in collaboration with implementation experts (5). These experts rated the importance and feasibility of compiling 73 implementation strategies to barriers categorized by the CFIR framework (5). The tool allows users to select the identified CFIR determinants. Hereafter, a list of relevant strategies is presented per identified determinant for implementation. For each strategy, the tool provides the percentage of experts who ranked that particular strategy in their top seven. This percentage can be interpreted at two levels of endorsed strategies, namely, *Level 1* endorsed ERIC strategies (i.e., more than 50% of the experts ranked this as one of their top seven strategies for that barrier) and *Level 2* endorsed ERIC strategies (i.e., between 20% and 50% of the experts ranked this as one of their top seven strategies for that barrier) (5, 23). The research group determined that, for the continuation of this study, the top three strategies with the highest agreement or three strategies with an agreement higher than 50% would be used.

Although the CFIR-ERIC Matching Tool provides a convenient global overview of appropriate implementation strategies, the ERIC taxonomy is not exhaustive, and additional efforts are needed to do justice to all identified barriers (24). The research group hypothesized that some barriers might be rooted in the specific behavior of healthcare professionals or older adults receiving the intervention and that these aspects were underrepresented in the ERIC taxonomy. Therefore, an additional literature search was conducted to incorporate behavioral change strategies. Implementation taxonomies and theories, including the taxonomy of Kok et al. (25), Greenhalgh et al. (26), and the Theoretical Domain Framework (17), were consulted to identify implementation strategies targeting behavior.

##### 2.4.2.2 Developing new strategies in codesign

In addition to selecting implementation strategies based on taxonomies and theories described in the literature, input from involved healthcare professionals was retrieved during two codesign sessions. Codesign sessions were scheduled with 10 healthcare professionals (physiotherapists and dieticians). In total, two 90-min online (due to the COVID restrictions) codesign sessions with healthcare professionals were held. At the beginning of the sessions, healthcare professionals were informed about ProMuscle through a short presentation. Under the supervision of a researcher, healthcare professionals discussed possible effective strategies to overcome barriers for implementing ProMuscle. To obtain full objectivity, healthcare professionals were unaware of the implementation strategies identified from the literature. In the end, if strategies from the literature were not mentioned by healthcare professionals, the researcher would propose them to the healthcare professionals to explore whether they could also be considered effective strategies.

##### 2.4.2.3 Triangulation

The strategies retrieved from both the literature and codesign sessions were described in a matrix. Where possible, the research group matched the strategies proposed by healthcare professionals to those from the literature and combined them into the matrix. The strategies that remained and could not be combined with the strategies from the literature were treated as new and added to the matrix.

#### 2.4.3 Step 3. Evidence for implementation strategies

Proctor et al. stated that providing theoretical justification for implementation strategies can address their potential working mechanisms, giving insight into how and why a strategy might facilitate change (2). Theoretical justification can take various forms: empirical, theory-based, and pragmatic (2).

*Empirical evidence* is considered evidence from research or an individual’s knowledge and experience with strategies that have been proven effective.

*Theory-based evidence* refers to the theoretical knowledge gained in a research field or concerning a specific subject.

*Pragmatic justification* is derived from clinical expertise, experiences, or the needs of relevant stakeholders concerning overcoming barriers. Although pragmatic evidence does not provide empirical or theoretical evidence for strategies, it can provide insights into the rationale for identifying factors that should be addressed and how strategies could address them (2, 27). In the context of the present study, the research group sought evidence for the identified implementation strategies in scientific literature. The literature that described theories and taxonomies linking specific implementation determinants to strategies was used. First, studies that investigated individual strategies were sought in the database of EPOC and implementation science journals. If the effectiveness of specific strategies was not examined in the literature, theory-based justification was sought in existing theories for the underlying constructs identified in step 1. Also, studies reporting implementation strategies in similar contexts were consulted.

In addition to seeking empirical evidence and theoretical justification, we aimed to derive pragmatic justification during the codesign sessions with healthcare professionals. Healthcare professionals discussed possible effective strategies to overcome barriers for implementing ProMuscle and provided insights into the effectiveness of the strategies based on their clinical expertise and needs. Also, pragmatic justification for the strategies was obtained during meetings with implementation experts and researchers, as well as through interviews with older adults, drawing on their experiences and needs.

#### 2.4.4 Step 4. Operationalizing implementation activities

The next step in developing appropriate implementation strategies involves operationalizing the implementation activities in full detail. The literature emphasizes the needs and challenges of specifying and reporting implementation strategies (2). Guided by the recommendations for specifying and reporting implementation strategies outlined by Proctor et al. (2), the operationalization of the implementation strategies considered seven dimensions: actor, action, action targets, temporality, dose, implementation outcomes addressed, and theoretical justification. These dimensions should be fully described to facilitate measurement and reproducibility.

With respect to the current study, a matrix was developed to describe all seven dimensions of each proposed implementation strategy.

For this step, codesign with stakeholders was established in an iterative way through consensus meetings with the research team, meetings with two implementation experts, and interactive work sessions with healthcare professionals, including physiotherapists and dieticians. During two 90-min codesign sessions, healthcare professionals were divided into groups. Across the sessions, five groups worked with themes containing several overlapping strategies to make sure all strategies had been covered and to limit workload per codesign session.

The matrix was continuously supplemented with input from healthcare professionals, implementation experts, and research groups during the sessions, resulting in a complete matrix that incorporated input from stakeholders and the literature.

#### 2.4.5 Step 4b. Development of an implementation toolbox

To meet the needs of professionals, implementation materials, in the form of an implementation toolbox, were developed (Implementation Mapping step 4). It was important to create a practical tool to assist healthcare professionals and provide them with the ability to tailor the implementation strategies to their specific context. As mentioned earlier, the research group was aware of the different settings in which ProMuscle would be implemented, consequently leading to different contexts and barriers.

During the development of the implementation toolbox, the research group consulted 1 implementation expert and 10 professionals to create a practical tool for healthcare professionals implementing ProMuscle. The described implementation activities were presented to an implementation expert. Also, based on the experiences of the experts, the most practical way to present the activities in an online platform was discussed. Moreover, the presentation of the tool was designed to be user-friendly and inviting for professionals to use it.

## 3 Results

### 3.1 Population

The research team, along with Dutch implementation experts (*n* = 2) and HCPs, i.e., physiotherapists (*n* = 8) and dieticians (*n* = 2) working in the Region Foodvalley, participated in the interactive codesign sessions to provide input for the development of implementation strategies. Table 1 presents the participation of stakeholders across each step.

**Table 1:** Participants of the codesign sessions presented for all four steps of the chosen Implementation Strategy Mapping Method.

Gender	Profession	Work experience (years)	Step 1	Step 2	Step 2*	Step 3	Step 4	Step 4b
	Research group		X	X	X	X	X	X
Female	Physiotherapist	13	X	X		X	X	
Female	Physiotherapist	6	X	X		X	X	
Male	Physiotherapist	37	X	X		X	X	
Female	Physiotherapist	14	X	X		X	X	
Male	Physiotherapist	16	X	X		X	X	
Female	Physiotherapist	39	X	X		X	X	
Female	Physiotherapist	4	X	X		X	X	
Female	Physiotherapist	8	X	X		X	X	
Female	Dietician	7	X	X		X	X	
Female	Dietician	25	X	X		X	X	
Female	Implementation expert					X		X
Female	Implementation expert							X

Step 1, identifying barriers and theoretical constructs; Step 2, linking strategies to barriers; Step 2*, assigning strategies to overarching themes; Step 3, evidence for strategies; Step 4, operationalizing implementation activities; Step 4b, development of an implementation toolbox.

### 3.2 Outcomes

**Figure 1:**
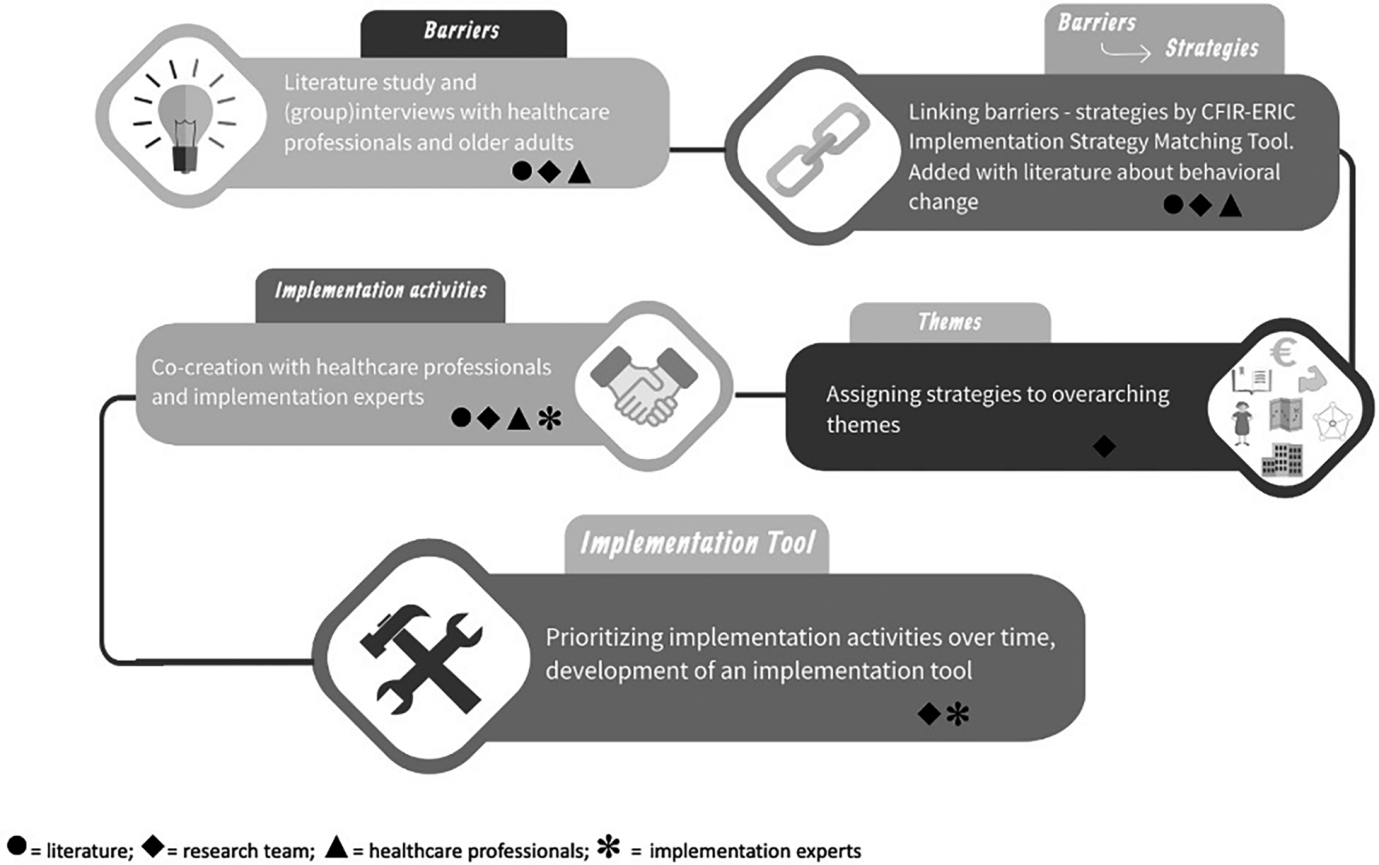
Flowchart development implementation strategy bundle and implementation toolbox, including methods used to retrieve input.

Implementation strategies for facilitating the implementation of ProMuscle in primary care were selected, described, and operationalized using four adapted steps of Implementation Mapping. In all four steps, different ways to engage stakeholders were included, as presented in the following. Figure 1 visualize the steps including the methods used to retrieve input and the involved stakeholders. Because of the fact that ProMuscle will be implemented in multiple settings, a significant number of barriers and linking strategies emerged. Therefore, an extra step, assigning strategies to themes, was added to step 2 (Figure 1).

#### 3.2.1 Step 1. Identifying barriers and theoretical constructs

In a previous study (15), determinants influencing the implementation of combined lifestyle interventions were identified through a literature review and interviews with relevant stakeholders. A total of 654 determinants were identified, representing all CFIR domains, that could influence the implementation of combined lifestyle interventions similar to ProMuscle (15). Relevant stakeholders like physiotherapists and dieticians validated and prioritized these determinants during interviews. This resulted in 10 main barriers for the implementation of a combined lifestyle intervention in primary care. The top 10 most common determinants are as follows: “other personal attributes,” “knowledge and beliefs about the intervention,” “readiness for implementation,” “network and communication,” “implementation climate,” “design quality and packaging,” “costs,” “patient needs and resources,” “cosmopolitanism,” and “engaging” (Table 2).

**Table 2:** Identified determinants influencing the implementation of combined lifestyle interventions linked to theoretical constructs.

Domain	Construct	Definition of the CFIR construct	Theoretical construct	Theory
Characteristics of individuals	Other personal attributes	A broad construct to include other personal traits such as tolerance of ambiguity, intellectual ability, motivation, values, competence, capacity, and learning style	Attitude	Theory of planned behavior, social cognitive,
			Self-efficacy	Social cognitive theory, TDF
			Skills	Implementation effectiveness, TDF
			Knowledge	TDF
Characteristics of individuals	Knowledge and beliefs about the intervention	Individuals’ attitudes toward and value placed on the intervention as well as familiarity with facts, truths, and principles related to the intervention	Attitudes	TDF
			Commitment	Implementation effectiveness model
			Knowledge	Social cognitive theory
Inner setting	Readiness for implementation	Tangible and immediate indicators of organizational commitment to its decision to implement an intervention	Commitment	Social cognitive theory
			Social norms	Implementation effectiveness model
			Resources	TDF
Inner setting	Network and communication	The nature and quality of webs of social networks and the nature and quality of formal and informal communications within an organization	Organizational commitment	TDF
Inner setting	Implementation climate	The absorptive capacity for change, shared receptivity of involved individuals to an intervention, and the extent to which use of that intervention will be rewarded, supported, and expected within their organization	Climate for implementation	Implementation effectiveness model, TDF
Innovation characteristics	Design quality and packaging	Perceived excellence in how the intervention is bundled, presented, and assembled	Intervention effectiveness	Implementation effectiveness model
			Resources	TDF
Innovation characteristics	Costs	Costs of the intervention and costs associated with implementing the intervention including investment, supply, and opportunity costs	Costs	Health beliefs model
			Incentives	Implementation effectiveness
			Resources	TDF
Outer setting	Patient needs and resources	The extent to which patient needs, as well as barriers and facilitators to meet those needs, are accurately known and prioritized by the organization	Incentives,	Implementation effectiveness
			Knowledge	TDF
			Motivation,	TDF
			Resources	TDF
Outer setting	Cosmopolitanism	The degree to which an organization is networked with other external organizations	Environmental, leadership	TDF
			Organizational commitment	TDF
			Social norms	Social cognitive theory
Process	Engaging	Attracting and involving appropriate individuals in the implementation and use of the intervention through a combined strategy of social marketing, education, role modeling, training, and other similar activities	Motivation	TDF, health belief model
			Incentives	TDF
Process	Innovation participants	Attract and encourage recipients to serve on the implementation team and/or participate in the innovation	Attitude	TDF
			Commitment	Implementation effectiveness
			Social support	TDF

TDF, theoretical domain framework.

These determinants were linked to theoretical constructs. Some theoretical constructs were similar for multiple determinants. Moreover, most determinants could be linked to multiple theoretical constructs. Table 2 presents all 10 determinants with underlying constructs. The models used to link the determinants to constructs were the theoretical domain framework (17), implementation effectiveness model (28), health belief model (29), and social cognitive theory (30).

#### 3.2.2 Step 2. Linking barriers to strategies

##### 3.2.2.1 Linking to existing taxonomies described in the literature

The selected constructs from CFIR in the previous step were entered in the CFIR-ERIC Matching Tool, and this method resulted in multiple strategies advised for the specific determinants. The initial step involves excluding strategies deemed not applicable because they did not align with the context for implementing ProMuscle. For example, the strategy to make billing easier was a level 2 endorsed strategy for the construct “costs.” However, the combined lifestyle intervention ProMuscle is not reimbursed, and recipients are required to pay for participation. Therefore, the research group decided that this strategy would not be suitable for implementation in this phase. However, if ProMuscle were to be reimbursed, this strategy could be considered and added to the strategy bundle if deemed necessary.

For constructs “patient needs and resources,” “engaging,” and “other personal attributes,” the CFIR-ERIC strategy matching tool did not yield (appropriate) strategies to align with the context. In the end, this step resulted in 40 appropriate implementation strategies. Of the 40 strategies, 32 were retrieved from the ERIC taxonomy (22), 5 from the TDF, and 2 from the taxonomy of Kok et al. (25).

##### 3.2.2.2 Developing new strategies in codesign

In addition, the input from healthcare professionals and implementation experts during the codesign sessions was mostly practical and was not specifically linked to implementation strategies as described in the literature and the ERIC taxonomy. The activities proposed by healthcare professionals align with the action dimension, according to Proctor et al. (2), for most of the strategies that were found in the literature (as presented in Table 3).

**Table 3:** Implementation strategies assigned to overarching themes and relating CFIR construct(s).

Theme	Strategy from taxonomies and theories	Actions proposed by HCPs in work sessions	Underlying CFIR domain—construct
Assessing the context	Conduct local needs assessment		Inner setting—readiness for implementation
			Outer setting—patient needs and recources
	Assess for readiness and identify barriers and facilitators		Inner setting—implementation climate
			Inner setting—readiness for implementation
Network internally	Build a coalition	Staff meetings	Inner setting—readiness for implementation
			Inner setting—network and communication
	Organize clinician implementation team meetings	Informing and promoting	Inner setting—network and communication
	Promote network weaving	Maintain collaboration	Inner setting—network and communication
Network externally	Promote network weaving	Informing and promoting	Outer setting—cosmopolitanism
	Develop academic partnerships		Outer setting—cosmopolitanism
	Build a coalition	Forming a network	Outer setting—cosmopolitanism
Costs	Access new funding	Access funding	Intervention characteristics—costs
	Alter incentive/allowance	Incentives for recipients	Intervention characteristics—costs
			Inner setting—implementation climate
	Develop resource-sharing agreements	Sharing knowledge, space, and materials	Intervention characteristics—costs
Knowledge	Develop educational materials	Education	Characteristics of individuals—knowledge and beliefs about the intervention
		Promotion materials and protocols	Intervention characteristics—design quality and packaging
			Process—engaging
	Conduct ongoing training	Yearly training	Characteristics of individuals—other personal attributes
	Conduct educational meetings	Frequent evaluations	Characteristics of individuals—knowledge and beliefs about the intervention
			Process—engaging
Champions	Identify and prepare champions	Champions	Characteristics of individuals—knowledge and beliefs about the intervention
			Inner setting—implementation climate
			Process—engaging
Patient needs and resources	Involve patients, consumers, and family members	Engaging older adults	Process—engaging innovation participants
			Outer setting—patient needs and resources
	Prepare patients/consumers to be active participants	Group coherence, personal approach	Process—engaging innovation participants
		Setting goals	Outer setting—patient needs and resources
		Coaching	
	Intervene with patients and consumers to enhance uptake and adherence	Share results with recipients	Process—engaging innovation participants
			Outer setting—patient needs and resources
	Promote adaptability	Intervention fitting the context	Outer setting—patient needs and resources intervention characteristics—design quality and packaging
	Obtain and use patients’/consumers’ and family feedback		Outer setting—patient needs and resources

For the strategies derived from the literature that were not mentioned by healthcare professionals, the researchers asked whether the remaining strategies could be effective or not. Three strategies that appeared in the literature but were not mentioned by healthcare professionals were “conduct local need assessment,” “assess for readiness and identify barriers and facilitators,” and “develop academic partnerships.” Because the healthcare professionals were not experienced in implementation science, and likely had insufficient awareness for assessing the context (needs, barriers, and facilitators), the research group decided to elaborate on these strategies anyway. Moreover, the three strategies were classified as level 1 strategies according to the CFIR-ERIC Matching Tool.

To illustrate the elaboration of this step, in the following box (Box 1), we present how the construct “costs” within the domain *intervention characteristics* was linked to implementation strategies.

BOX 1 **Linking construct “costs” to implementation strategies with the CFIR-ERIC tool**

Entering determinant “costs” (intervention characteristics) into the CFIR-ERIC tool resulted in the following strategies: “access new funding” (72%), “alter incentives” (44%), and “develop resource sharing agreements” (32%). Also, for construct *implementation climate*, strategy “alter incentives” was presented. This outcome suggested that a single strategy could address multiple barriers.

##### 3.2.2.3 Triangulation: assigning strategies to overarching themes

The literature search and consultation with healthcare professionals revealed a great number of strategies. During consultation with the research group, it was noticed that some implementation strategies were applicable to multiple determinants. Therefore, it was hypothesized that some strategies would affect multiple barriers. In addition, the large number of strategies could burden healthcare professionals (31). As a result, the research group aimed to identify overarching themes within the strategies and introduced an extra step within the adopted version of Implementation Mapping. A total of four consensus meetings were conducted with the research group to provide an overview, create overarching themes, and assign strategies to the themes. Ultimately, 20 unique strategies were assigned to 7 overarching themes: *assessing the context*, *network internally*, *network externally*, *costs*, *knowledge*, *champions*, and *patient needs and resources*. Table 3 presents the seven themes, providing a complete overview of strategies derived from the literature and input from healthcare professionals, along with the constructs to which these strategies were linked. Appendix A provides a description of the constructs that eventually fell under the themes. Box 2 presents the description of the theme *costs.*

BOX 2 **Description of constructs that felt under theme *costs***

*Costs*: This theme primarily reflects on construct *intervention characteristics*. Also, the construct *implementation climate* is related to this theme, as insufficient time (and money) for the implementation process itself was identified as a barrier for implementation.

#### 3.2.3 Step 3. Evidence for implementation strategies

Eventually, this third step resulted in justification for every strategy, which is extensively described in Appendix B. Empirical evidence was found for activities within the following strategies: “assess for readiness and identify barriers and facilitators” (32, 33), “build a coalition” (34), “conduct ongoing training” (35), “conduct educational meetings” (34, 36), and “intervene with patients and consumers to enhance uptake and adherence” (35). Most strategies could be justified by underlying theoretical constructs or models mostly based on organizational change (19, 20, 37), system change (34, 38–40), and behavior change (17, 25, 41, 42). Also, during the codesign sessions, healthcare professionals and implementation experts provided pragmatic evidence from their own experience with implementation, as well as based on their needs. In previous research, older adults were interviewed, which resulted in pragmatic evidence for implementation strategies concerning the strategies in theme “Patient needs and resources”.

To illustrate the improved methodology of developing implementation strategies, the theme *costs* will be described in detail in Box 3. For a complete description of all strategies, including the evidence for each strategy, see Appendix B.

BOX 3 **Description of the theoretical justification of the strategies in theme *costs***

For theme *costs*, no empirical evidence was found for the three strategies in the EPOC database and implementation journals in similar contexts. This is probably because the insurance and funding possibilities in the Netherlands differ from those in, for example, the United States where most implementation strategy effectiveness studies are conducted. However, Greenhalgh et al. (26) presented several studies where funding contributed to the success or failure of implementation.

Therefore, literature was sought within existing theories and models. The used taxonomies of Michie et al. (17) and Kok et al. (25) did not provide relevant references. The research group conducted a search for studies addressing “funding possibilities,” “alter incentives,” and “ sharing recourse agreements” and their possible underlying theories or working mechanisms for implementation. A review from Dopp et al. was found (40), where strategies concerning funding an EPB implementation were discussed. Dopp et al. highlighted that funding is necessary to cover the costs of care. Grants serve as a means to reimburse the EBPs and incentivize their use (40). Covering the costs leads to decreased expenses for service providers, which ultimately can increase the acceptability of the EBP. Moreover, incentives provide resources (e.g., training, consultation) that may be difficult to purchase for health services.

Consequently, the literature search resulted in theoretical justification for the strategies “assess new funding” (40) and “alter incentives” (34, 40). Healthcare professionals provided practical activities and practice-based, pragmatic justification for the strategies “alter incentives,” “develop resource sharing agreements,” and “assess funding possibilities.”

During the work sessions, healthcare professionals mentioned that costs could be one of the main barriers for implementation. Because the combined lifestyle intervention ProMuscle is not reimbursed by healthcare insurance, older adults, especially those with little financial possibilities, may be unable to participate. Moreover, the costs of the program could also impact the recruitment of older adults. This could be due to the limited knowledge of older adults about the benefits of a program like ProMuscle. Healthcare professionals stated that assessing funding possibilities and informing older adults about the benefits of ProMuscle could contribute to optimal recruitment and adoption.

For theme *costs*, healthcare professionals proposed several implementation activities focused on the costs of the intervention and the time spent on implementation by professionals.

The importance of these activities was highlighted by professionals’ experiences. Healthcare professionals expressed that it is important for possible participants to know what to expect and to prevent dropouts due to (unexpected) costs. In addition to implementation activities concerning the costs of delivering ProMuscle (access funding possibilities), healthcare professionals also provided insight into what they needed to be able to implement ProMuscle in their practice (alter incentives). Deliberating with the manager of their practice to make time for implementing the intervention was mentioned as crucial to be able to evaluate, upscale, and sustain the implementation. Also, practical incentives such as promotion materials, protocols, and templates were mentioned as needs by healthcare professionals. Finally, using the current implementation group to exchange knowledge, materials, and even workplace was mentioned (develop resource sharing agreements). Having the ability (time, materials, and facilities) to implement the intervention and ensuring that fellow implementers will be open-minded in sharing resources ensures that healthcare professionals in their network are on the same page. According to healthcare professionals, being on the same page and uniformly delivering the intervention could enhance the success of implementation.

#### 3.2.4 Step 4. Operationalizing implementation activities

The research group translated the retrieved strategies into Dutch and provided global information about the strategies to further operationalize them during the codesign sessions.

The first group of healthcare professionals worked with theme *costs*, and the second group worked with themes *process*, *intervention*, and *knowledge*. The third group worked with themes *network internally*, *network externally*, and *patient needs and resources*. The fourth group worked with theme *knowledge*. A fifth group consisting of dieticians was considered a validation group because the other four groups consisted of physiotherapists. The group of dieticians checked whether they agreed with the proposed activities and were asked if they missed specific activities.

Healthcare professionals provided additional and practical input concerning the “actors,” “action,” “dose,” and “justification” dimensions, according to Proctor et al. (2).

The research group complemented the specification with input from the literature. Input from the literature, research groups, and healthcare professionals resulted in fully detailed implementation strategies for all seven themes. A complete description of the strategies for theme *costs* is presented in Table 4. For the remaining themes, the strategies are described in Appendix B.

**Table 4:** Description of strategies concerning the theme costs following the recommendation of Proctor et al. (2).

Name of the strategy	CFIR domain—construct affected	Definition of the strategy	The actors	The action	The targets	Temporality	Dose	Implementation outcome affected	Justification empirical evidence, theory-based evidence, pragmatic justification
Access new funding	Intervention characteristics—costs	Access new or existing money to facilitate the implementation	HCP	Writing calls or granting applications to cofinance the intervention	Possible funders of the intervention are healthcare insurance, municipalities, funds, etc. Cofunding gives an opportunity to lower the costs of the intervention for recipients	Ideally, before the start of the implementation but can also be conducted throughout the implementation	Depending on the temporality of the funding, new funding can be assessed as many times as needed	Feasibility, adoption, fidelity, acceptability	Pragmatic from experiences of healthcare professionals and theoretical evidence (40)
Alter incentive/allowance	Intervention characteristics—costs Inner setting—implementation climate	Work to incent the adoption and implementation of clinical innovation	HCP RG	Researchers will inform healthcare professionals about the implementation and the intervention and healthcare professionals will discuss with their manager for extra time to set up and roll out the implementation	Improving knowledge of healthcare professionals about the need for facilities during implementation and increasing the opportunity from implementing, facilitated by managers of healthcare professionals	Before the start of the implementation and and during the implementation, if necessary	A 30-min phone call/online meeting between the researcher and healthcare professionals; during the implementation, monthly evaluation meetings are held; healthcare professionals will discuss (once) the opportunity for extra time with their managers before the start of the implementation	Feasibility, fidelity, adoption	Theory-based (34, 40)
Develop resource-sharing agreements	Intervention characteristics—costs	Develop partnerships with organizations that have the resources needed to implement the innovation	HCP	Healthcare professionals reach out to each other to share information, knowledge, and materials	Improving knowledge and facilities of healthcare professionals implementing ProMuscle	Before, during, and after the implementation	Healthcare professionals contact each other if necessary, before, during, or after the implementation	Feasibility, fidelity, sustainability	Pragmatic from experiences of healthcare professionals

HCP, healthcare professional; RG, research group.

Themes *Assessing the context* and *champions* were seen as important for all other themes. Therefore, the strategies assigned to these themes were considered obligatory to start the implementation.

#### 3.2.5 Step 4b. Development of an implementation toolbox

The research group consulted multiple implementation experts and professionals to create a practical tool for healthcare professionals implementing ProMuscle**.** Implementation experts mentioned that it was important for the tool to be easy to use. It should not take much time to understand the tool. They emphasized the importance of providing an overview where professionals should not have to perform extensive scrolling. Also, the implementation activities should be presented in chronological order, rather than by theme.

Therefore, the research team assigned every implementation activity to a specific time frame. The activities could be assigned to one or more time frames. The following time frames were used: 8–6 weeks preimplementation, 6–4 weeks preimplementation, 4–0 weeks preimplementation, implementation, and sustainment. This resulted in an online implementation toolbox in which implementation actions are chronologically described and bundled per theme. In this way, healthcare professionals are free to choose which theme would apply to their specific context. Moreover, a function was built to check whether actions were conducted and to add remarks.

The four steps resulted in a full description of 20 strategies, divided over 7 overarching themes. A complete description of all 20 strategies and the barriers they address is presented in Appendix B. The theory-based and practical implementation activities were added to a web-based implementation tool. Figure 1 presents an overview of the conducted steps and the methods used to retrieve input. As shown in Figure 1, the steps of Implementation Mapping were slightly changed, and an extra step (themes) was added. Moreover, in every step, relevant stakeholders provided input to provide an implementation strategy bundle for healthcare professionals that can be tailored to their specific contexts, and added this bundle in an online toolbox.

## Discussion

This paper describes the methodology of developing a theoretically justified and practically tailored implementation strategy bundle to implement a combined lifestyle intervention for community-dwelling older adults across multiple settings in primary care. The Implementation Strategy Mapping Method was guided by Implementation Mapping (9). Initially, the four steps of Implementation Mapping were followed. Because this study focuses on multiple settings in primary care and various contexts were explored, a great number of determinants for implementation emerged, which ultimately led to 40 linked implementation strategies. The addition of an extra step to the methodology was deemed necessary to provide structure to the array of implementation strategies. Moreover, the diverse collection of strategies could enable healthcare professionals to tailor their strategies according to their specific contexts.

Ultimately, the structural approach guided by Implementation Mapping and the embedded codesign with healthcare professionals and implementation experts led to the development of a practical and theory-informed strategy bundle. Through codesign, the strategies were tailored to the context in which they were supposed to be applied. The implementation toolbox serves as a guide for healthcare professionals, assisting them during the implementation and overcoming barriers related to their contexts.

A large number of implementation strategies, totaling 20, were described in detail and included in the final implementation toolbox for healthcare professionals who aim to implement a combined lifestyle intervention. The large proportion of strategies can be justified by the multiple determinants that were found as possible barriers for implementing a combined lifestyle intervention. For the implementation of a combined lifestyle intervention, determinants at multiple levels can affect the implementation results. Therefore, by including multiple strategies in the implementation toolbox, we can ensure that healthcare professionals can tailor strategies aligning with their specific contexts and can adjust them when encountering other barriers during the implementation process.

The inclusion of the extra step *assigning strategies to overarching themes* in the development of the implementation strategy was prompted by the perceived burden for healthcare professionals. Creating themes resulted in strategy bundles relating to the specific themes. Multiple studies present the development and use of multicomponent strategies (6, 7, 43, 44). Moreover, the use of multicomponent strategies is highlighted by Cooper et al. (45), where various combinations of strategies were found effective for sustaining the implementation of an EBP (4, 43). The wide use of multicomponent strategies in implementation science, and the ones that were investigated and found effective in different trial studies, is grounded in the understanding that implementation is often influenced not only by one determinant but by a combination of determinants.

Moreover, the context in which an intervention is implemented greatly influences the success of implementation (46). Therefore, as addressed by Nilsen et al. (46), the difference in contexts highlights the importance of tailoring the implementation to specific contexts. This is supported by a Cochrane review in which it was found that tailored implementation strategies were more effective than non-tailored strategies (47, 48).

Because this study described strategies for multiple barriers, an implementation plan can be tailored to the specific contexts in which the intervention is implemented (6). In addition, due to input from healthcare professionals, actions for the strategies are very practical and should be applicable to (mostly) every healthcare practice implementing ProMuscle. Also, determinants for all levels of implementation according to the CFIR were considered in developing the implementation toolbox. Therefore, tailoring an implementation plan to specific contexts should be possible.

This paper not only addresses the development but also gives a transparent and complete description of the developed implementation strategies. It is not entirely surprising that most studies lack a description of the selection and development of implementation strategies and stakeholder engagement; developing strategies following one of the Implementation Strategy Mapping Methods is very time-consuming. However, because the strategies are detailed and based on theory and practice, fellow implementers can use this overview of a strategy bundle (Appendix B) in similar implementation processes of combined lifestyle interventions. Future research should focus on the working mechanism (49) of the implementation strategies developed in this study. With the results of this study, knowledge about the strategies could be used to implement other combined lifestyle interventions for community-dwelling older adults. If the implementation toolbox is found effective, it can be more widely deployed, adjusted to other contexts, or investigated for other interventions.

A strength of this study is that an Implementation Strategy Mapping Method by way of Implementation Mapping (9) was used to guide the process of developing implementation strategies. Implementation Mapping is considered a powerful approach because of its collaborative nature (43), which is perceived as critical in implementation (50). In the case of ProMuscle, where multiple barriers were identified that could influence the implementation of a combined lifestyle intervention, it could be suggested that multiple strategies are needed. But also, that for every setting, different (combinations) of strategies are appropriate. Therefore, other Implementation Strategy Mapping Methods could also be used as guidance for the development of the implementation strategy bundle, for example concept mapping, focus groups or conjoint analysis (8). However, because of the novelty of the research area in implementation strategy development models, little is known about the effectiveness of the models regarding the adoption of the implemented intervention (8). Therefore, we used Implementation Mapping, the most well-known and widely used method that incorporates stakeholder input, and adjusted its steps to better align with the scope of our study (multiple settings).

Another strength is the incorporation of codesign with stakeholders during the identification of determinants and the development of the strategies. Codesign was a great contributor to tailor strategies to the specific context of implementing a combined lifestyle intervention in primary care (1). For developing implementation strategies to implement ProMuscle, it was hypothesized that codesign would be beneficial for the fidelity and feasibility of the strategies and the alignment with the context. Also, stakeholder engagement is an effective way to engage healthcare professionals in further implementation and involvement in the implementation trials (11). The codesign sessions were an organic and iterative process during all four steps. During the codesign sessions, healthcare professionals provided input on possible actions concerning the seven themes. These codesign sessions provided practical input, and all proposed activities could be linked to implementation strategies suggested by the CFIR-ERIC tool and other taxonomies. Moreover, the codesign sessions resulted in tailored implementation strategies for all seven themes. Finally, the correspondence between the results of the literature search and the codesign sessions suggests that the developed implementation strategies match the context in which ProMuscle will be implemented.

A limitation of this study was that the CFIR-ERIC Matching Tool was used to identify strategies for the potential barriers. Although the CFIR-ERIC tool is widely used in implementation science, it is based on the experiences of implementation researchers and not all strategies included in the tool are evaluated for their effectiveness (22). However, this limitation was partly resolved by the literature search conducted in step 3. Although little empirical evidence was found for individual strategies, the justification lies in the theory and models underpinning the strategies to overcome specific barriers when implementing a combined lifestyle intervention. Further research should investigate not only the link between determinants and strategies but also the effectiveness of the bundled implementation strategies.

## Conclusion

The utilization of an Implementation Strategy Mapping Method, with an important role for codesign in each step, led to the development of theoretically justified and practical implementation strategies to support healthcare professionals to implement a combined lifestyle intervention for community dwelling older adults. A significant number of implementation strategies are fully described and can serve as a first overview for other implementers. The structural method, taking the context into account by incorporating codesign in all four steps, has resulted in a theoretically informed final product, an implementation toolbox. Therefore, the implementation toolbox could be a practical tool that can be tailored to an individual's context for healthcare professionals willing to implement a combined lifestyle intervention such as ProMuscle. Future research will focus on evaluating the implementation strategy bundle, including the implementation toolbox, regarding the implementation ProMuscle in primary care.

## Data Availability

The raw data supporting the conclusions of this article will be made available by the authors without undue reservation.

## Appendix A Description of constructs that are related to the themes

*Assessing the context:* Conducting local needs assessment was described as a strategy for the internal and external contexts. In addition, assessing readiness and identifying barriers and facilitators were strategies that align with theme *internal context*.

*Network internally:* Building a coalition, promoting network weaving, and organizing implementation team meetings were strategies assigned to theme *network internally*. The theme reflects mostly on the CFIR construct network and communication. However, the construct readiness for implementation also aligns with the theme with the corresponding strategy, “build a coalition.”

*Network externally*: Cosmopolitanism, e.g., working with other organizations, is the only construct assigned to theme *network externally*. Strategies concerning *network externally* were relatively similar to those concerning *network internally*. However, it is executed in different levels of the context and focuses on building and enhancing external collaboration with stakeholders. Therefore, actions described for the strategies in theme *network externally* are different from those described for *network internally*.

*Costs*: Theme *costs* reflect mostly on the construct intervention characteristics. Also, construct *implementation climate* is related to this theme, as insufficient time (and money) for the implementation process itself was identified as a barrier to implementation.

*Knowledge:* Strategies concerning theme *knowledge* reflect multiple levels within the implementation. Actions related to knowledge, such as materials, are described for stakeholders, recipients of the intervention, and healthcare professionals delivering the intervention. The strategies in theme *knowledge* relate to constructs “knowledge and beliefs about the intervention,” “design quality and packaging,” “engaging,” and “other personal attributes.”

*Champions:* Theme *champions* was linked to one strategy, addressing three constructs. Within the description of the strategies, a champion is mostly named as an actor. Because champions were named as actors for a great number of strategies and were found to have a major role in implementation, the theme *champions* should be incorporated into the other themes.

*Patient needs and resources*: More strategies addressed patient needs compared to the other themes. Most strategies in this theme were derived from other literature works concerning behavioral change. Moreover, many healthcare professionals proposed strategies that could be integrated into the intervention, for example, setting goals and motivational interviewing.

## Appendix B Description of implementation strategies following Proctor et al.’s recommendation for specifying and reporting implementation strategies.

**Table:**

Theme	Name of the strategy	CFIR domain—construct affected	Definition of the strategy	Actors	Action	Targets	Temporality	Dose	Implementation outcome affected	Justification empirical evidence, theory-based evidence, pragmatic justification
Assessing the context	Conduct local needs assessment	Inner setting—readiness for implementation	Collect and analyze data related to the need for the innovation; this assessment could be focused on the description of usual care and its distance from evidence-based care, outcomes of usual care, opinions from stakeholders on the need for an innovation or on special considerations for delivering the innovation in the local context	RG	Researchers develop a readiness tool to assess the different contexts of professionals to assess whether all required materials are available	Identifying the needs of physiotherapists and dieticians implementing ProMuscle	Before the start of the implementation	One questionnaire before the start of the implementation; completing the questionnaire takes up to approximately 10 min	Acceptability, appropriateness	Theory-based (35)
	Assess for readiness and identify barriers and facilitators	Inner setting—implementation climate Inner setting—readiness for implementation	Assess various aspects of an organization to determine its degree of readiness to implement, barriers that may impede implementation, and strengths that can be used in the implementation effort	RG	Assessment of barriers and facilitators through literature review and consultation with stakeholders; develop the readiness tool based on the previously identified barriers and facilitators; and assess the readiness tool with HCPs	Identifying barriers and facilitators of physiotherapists and dieticians for implementation of ProMuscle	Before the start of the implementation	One questionnaire before the start of the implementation; completing the questionnaire takes approx. 10 min	Appropriateness, feasibility	Empirical (30, 31), theory-based (26)
	Conduct local needs assessment	Outer setting—patient needs and resources	Collect and analyze data related to the need for innovation	HCP	Assessment of the needs of recipients of the intervention to align with the knowledge and needs in the first week of the intervention	Identify the needs of possible recipients of ProMuscle	Before the start of the intervention	During the intake of the intervention	Fidelity, patient centeredness	Pragmatic from experiences of HCPs
Costs	Access new funding	Intervention characteristics—costs	Access new or existing money to facilitate the implementation	HCP	Writing calls or grant applications to cofinance the intervention	Possible funders of the intervention are healthcare insurance, municipalities, funds, etc.; cofunding gives an opportunity to lower the costs of the intervention for recipients	Ideally, before the start of the implementation, but it can also be conducted throughout the implementation	Depending on the temporality of the funding; new funding can be assessed as many times as needed	Feasibility, adoption, fidelity, acceptability	Pragmatic from experiences of HCPs, theoretical (38)
	Alter incentive/allowance	Intervention characteristics—costs Inner setting—implementation climate	Work to incent the adoption and implementation of clinical innovation	RG HCP	Researchers will inform HCPs about the implementation and the intervention. HCPs will discuss with their manager for extra time to set up and roll out the implementation	Improving knowledge of HCPs about the need for facilities during implementation and increase the opportunity from implementing, facilitated by managers of HCPs	Before the start of the implementation and during the implementation, if necessary	A 30-min phone call/online meeting between the researcher and HCPs; during implementation, monthly evaluation meetings are held; HCPs will discuss (once) the opportunity for extra time with their managers before the start of the implementation	Feasibility, fidelity, adoption	Theory-based (32, 38)
	Develop resource-sharing agreements	Intervention characteristics—costs	Develop partnerships with organizations that have the resources needed to implement the innovation	HCP	HCPs reach out to each other to share information, knowledge, and materials	Improving knowledge and facilities of HCPs implementing ProMuscle	Before, during, and after the implementation	HCPs contact with each other, if necessary, before, during, or after the implementation.	Feasibility, fidelity, sustainability	Pragmatic from experiences of HCPs
Network internally	Build a coalition	Inner setting—readiness for implementation	Recruit and cultivate relationships with partners in the implementation effort	HCP	Assessing and analyzing which colleagues are necessary for the implementation. Start a network or join an existing network within the organization to inform and collaborate about the implementation of ProMuscle	Involving HCPs and stakeholders within the organization in the implementation of ProMuscle	Before, during, and after the implementation	Assessing and analyzing stakeholders before the implementation; participate in networks and inform colleagues frequently (once a month depending on the original organization structure)	Adoption, sustainability	Empirical (32), theory-based (40)
	Organize clinician implementation team meetings	Inner setting—network and communication	Develop and support teams of clinicians who are implementing the innovation and give them protected time to reflect on the implementation effort, share lessons learned, and support one another’s learning	RG CH (HCP)	Set up a project team consisting of colleagues involved in the intervention; conduct monthly meetings containing evaluation moments concerning the implementation of ProMuscle	Involving HCPs with the implementation and increasing knowledge about the implementation and intervention.	Before, during, and after the implementation	Set up a project team before the implementation, conduct monthly meetings during the implementation, and maintain the involvement after the implementation	Adoption	Theory-based (37)
	Promote network weaving	Inner setting—network and communication	Identify and build on existing high-quality working relationships and networks within and outside the organization, organizational units, teams, etc. to promote information sharing, collaborative problem-solving, and a shared vision/goal related to implementing the innovation	CH	Analyzing whether networks regarding older adults in community care already exist within the organization; join and, if necessary, let other parties join the network who are considered important for the implementation of ProMuscle; stimulate participants of the network to collaborate and share recourses and information about the intervention	Involving HCPs with the implementation and increasing knowledge about the implementation and intervention.	Before, during, and after the implementation	Analyze current networks before the implementation, join networks during the implementation, and maintain the involvement after the implementation	Adoption, sustainability	Theory-based (36)
Network externally	Promote network weaving	Outer setting—cosmopolitanism	Identify and build on existing high-quality working relationships and networks within and outside the organization, organizational units, teams, etc. to promote information sharing, collaborative problem-solving, and a shared vision/goal related to implementing the innovation	CH HCP	Analyzing what networks regarding older adults in community care already exist; join and, if necessary, let other parties join the network who are considered important for the implementation of the intervention; stimulate participants of the network to collaborate and share resources and information about the intervention	Involving relevant stakeholders with the implementation of ProMuscle. Engage stakeholders by providing information about the intervention during meetings	Before, during and after the implementation	Analyze current networks before the implementation, join networks during the implementation, and maintain the involvement after the implementation	Adoption, sustainability	Theory-based (36)
	Develop academic partnerships	Outer setting—cosmopolitanism	Partner with a university or academic unit for shared training and bringing research skills to an implementation project	CH HCP	Contact research institutes like universities. Involve the institutes with the implementation. Explore if there are possibilities to be supported by the institute during the implementation	Involve and engage researchers from research institutes; create resource facilities for the implementation	Before, during, and after the implementation	Contact institutes before implementation, remain in contact, and share resources/collaborate during and after the implementation	Acceptability, adoption	Pragmatic (49)
	Build a coalition	Outer setting—cosmopolitanism	Recruit and cultivate relationships with partners in the implementation effort	CH HCP	Assessing and analyzing appropriate and necessary stakeholders from external institutions for the implementation; start a network or join an existing network to inform and collaborate about the implementation of ProMuscle	Involving relevant external stakeholders (e.g., general practitioners, community workers, and policy officers) with the implementation of ProMuscle; Engaging stakeholders by providing information about the intervention during meetings	Before, during, and after the implementation	Assessing and analyzing stakeholders before the implementation; participating in networks and informing stakeholders frequently depending on agreements about contact	Adoption, sustainability	Theory-based (37)
Knowledge	Develop educational materials	Characteristics of individuals—knowledge and beliefs about the intervention Intervention characteristics—design quality and packaging	Develop and format manuals, toolkits, and other supporting materials in ways that make it easier for stakeholders to learn about the innovation and for clinicians to learn how to deliver the clinical innovation	CH HCP	Researchers develop educational material and courses for HCPs; HCPs prepare materials to inform recipients and stakeholders about the intervention (flyers, presentations, etc.)	Improving opportunities and capabilities of HCPs executing the intervention, stakeholders, and recipients of ProMuscle	Before, during, and after the implementation	Develop educational materials for the HCPs before the implementation; HCPs further develop materials during the implementation	Acceptability, adoption, feasibility, fidelity	Theory-based (14, 50), pragmatic from needs of HCPs
	Conduct ongoing training	Characteristics of individuals—other personal attributes	Plan for and conduct training in clinical innovation in an ongoing way	RG IO	One year after the first ProMuscle course, organize a follow-up meeting to get back on the course, content, and background information of ProMuscle	Improving and maintaining knowledge, skills, and capabilities of HCPs delivering ProMuscle	Before, during, and after the implementation	Before the first HCPs start delivering the intervention, they follow the ProMuscle course; during the implementation, meetings are held to keep HCPs involved; every 1 year after the implementation, HCPs follow a follow-up course	Acceptability, adoption	Empirical (33), theory-based (14, 22)
	Conduct educational meetings	Characteristics of individuals—knowledge and beliefs about the intervention	Hold meetings targeted toward different stakeholder groups to teach them about clinical innovation	RG IO	Assessing involved stakeholders and continued intervention meetings to inform stakeholders about the implementation of the intervention	Engaging stakeholders (e.g., HCPs, general practitioners, community workers, and policy officers) by involving and informing them about the intervention.	Before, during, and after the implementation	Before the implementation, set up a network of involved stakeholders; During and after the implementation, continue meeting with the network	Acceptability, sustainment	Empirical (32, 34), theory-based (22), pragmatic
Champions	Identify and prepare champions	Characteristics of individuals—knowledge and beliefs about the intervention Inner setting—implementation climate	Identify and prepare individuals who dedicate themselves to supporting, marketing, and driving through an implementation, overcoming indifference or resistance that the intervention may provoke in an organization	RG and HCP	Within every healthcare practice willing to implement ProMuscle, a champion will be identified. The researchers will provide information about the tasks of a champion through an online guide	To make sure that within an organization, at least one healthcare professional acts as a champion to facilitate the adoption of ProMuscle	Before the implementation	Before the implementation, organizations can point out a champion. The champions are informed about their tasks by the research team	Adoption, fidelity	Pragmatic from the needs of HCPs and experience of implementation experts
Patient needs and resources	Involve patients, consumers, and family members	Process—engaging innovation participants Outer setting—patient needs and resources	Engage or include patients/consumers and families in all phases of the implementation effort, including training in the clinical innovation and advocacy related to the innovation effort	HCP	Recipients will be interviewed to inform possible barriers and facilitators to participate in ProMuscle. HCPs will inform recipients about the progress during the meetings and stimulate group adherence during the intervention	Make sure that the intervention aligns with the needs of the recipients of ProMuscle	Before the implementation and during the intervention	Before the implementation, barriers and facilitators will be identified. During the intervention, recipients will be informed and stimulated	Acceptability	Theory-based (51), pragmatic from experiences of HCPs and needs of recipients of ProMuscle
	Prepare patients/consumers to be active participants	Process—engaging innovation participants Outer setting—patient needs and resources	Prepare patients/consumers to be active in their care, to ask questions, and specifically to inquire about care guidelines, the evidence behind clinical decisions, or available evidence-supported treatments	HCP	HCPs will motivate recipients during the intervention with motivational interviewing, goal setting, participation, and/or shifting focus	Make sure that recipients participate and remain motivated during ProMuscle	During the intervention	In every consult/training during the intervention	Fidelity	Theory-based (22, 39), pragmatic evidence from experiences of HCPs and needs of recipients
	Intervene with patients and consumers to enhance uptake and adherence	Process—engaging innovation participants Outer setting—patient needs and resources	Intervene with patients/consumers to increase uptake of and adherence to clinical treatments. This includes consumer/patient reminders and financial incentives to attend appointments	HCP	Sending reminders and extra information to recipients through email/phone and motivational conversations during the intervention	Recipients of ProMuscle	During the intervention	In every consult/training, motivational talks will be held. Before every appointment, a reminder of the appointment will be sent. Once/twice a week, extra information will be sent per email. Information will be sent after completion of the program; the end date is advised to be after at least 1 year	Fidelity	Empirical (33), pragmatic from experiences of HCPs
	Promote adaptability	Outer setting—patient needs and resources Intervention characteristics—design quality and packaging	Identify the ways a clinical innovation can be tailored to meet local needs and clarify which elements of the innovation must be maintained to preserve fidelity	RG IO HCP	Assess the adaptability of ProMuscle; investigate which elements can be adjusted and which elements should be maintained; measure patient outcomes and collect adaptions during the implementation; tailor the intervention to patient needs	Make sure HCPs can match the intervention to the characteristics of recipients without losing the working elements of the intervention	Before, during, and after the implementation	Before implementation, ProMuscle will be discussed with HCPs to assess which elements they can deliver and what elements are evident. The literature will be gathered about the working elements	Fidelity, appropriateness	Theory-based (22), pragmatic from experiences of HCPs and researchers
	Obtain and use patients/consumers and family feedback	Outer setting—patient needs and resources	Develop strategies to increase patient/consumer and family feedback on the implementation effort	RG HCP	Assessment of effective ways to engage recipients of ProMuscle during interviews with older adults; HCPs give recipients possibilities to give feedback on the intervention and implementation	Make sure that the recruitment and the intervention align with the needs of recipients	Before implementation and during intervention	Before implementation, researchers will interview older adults to assess effective ways to engage possible recipients of ProMuscle and share this with HCPs. During the intervention, HCPs will ask recipients for feedback about the implementation process during the training/consultation and, if necessary, adapt the intervention to their needs	Implementation	Pragmatic

RG, research group; HCP, healthcare professional; CH, champion; IO, intervention owner.
